# The McGill University Health Centre Cancer Pain Clinic: A Retrospective Analysis of an Interdisciplinary Approach to Cancer Pain Management

**DOI:** 10.1155/2016/2157950

**Published:** 2016-03-31

**Authors:** Jordi Perez, Sara Olivier, Emmanouil Rampakakis, Manuel Borod, Yoram Shir

**Affiliations:** ^1^Cancer Pain Clinic, Division of Supportive and Palliative Care, McGill University Health Centre, Montreal, QC, Canada; ^2^Alan Edwards Pain Management Unit, McGill University Health Centre, Montreal, QC, Canada; ^3^JSS Medical Research, Saint Laurent, QC, Canada

## Abstract

*Context*. The McGill University Health Center (MUHC) Cancer Pain Clinic offers an interdisciplinary approach to cancer pain management for patients. The core team includes a nurse clinician specialist in oncology and palliative care, a palliativist, an anaesthetist, and a radiation oncologist. This tailored approach includes pharmacological and nonpharmacological therapies offered concurrently in an interdisciplinary fashion.* Objectives*. Description of the interdisciplinary MUHC cancer pain approach and analysis of treatments and outcomes.* Methods*. A retrospective analysis of new outpatients completing two subsequent visits (baseline and follow-ups: FU_1_, FU_2_) was conducted. Variables included (a) symptom severity measured by the Edmonton Symptom Assessment Scale, (b) pain and disability measured with the Brief Pain Inventory, and (c) analgesic plan implementation including pharmacological and nonpharmacological therapies.* Results*. 71 charts were reviewed. Significant pain relief was achieved consistently at FU_1_ and FU_2_. The average pain severity decreased by 2 points between initial assessment and FU_2_. More than half (53%) of patients responded with a pain reduction greater than 30%. Severity of other symptoms (i.e., fatigue, nausea, depression, and anxiety) and disability also decreased significantly at FU_2_. The total consumption of opioids remained stable; however, the consumption of short acting preparations decreased by 52% whereas the prescription of nonopioid agents increased. Beyond drug management, 60% of patients received other analgesic therapies, being the most common interventional pain procedures and psychosocial approaches.* Conclusion*. The MUHC interdisciplinary approach to cancer pain management provides meaningful relief of pain and other cancer-related symptoms and decreases patients' disability.

## 1. Introduction

Cancer pain is often cited as one of the most feared complications in cancer patients [1] and can occur as a result of the disease and/or its treatment. While the majority of cancer pain patients can be effectively treated using the World Health Organization guidelines, 10–20% fail to respond to conventional treatment [2].

*Justification for Interdisciplinary Approaches*. The assessment and treatment of cancer pain can be challenging and require the expertise of different clinical specialties. An anesthetist-led multidisciplinary program demonstrated initial and prolonged pain relief [3]. Similar positive results were described with a pharmacist-led cancer pain clinic [4]. On the other hand, poorer results were described after initial palliative care consultation [5]. Interestingly, authors suggested frequent follow-ups, phone calls, and collaboration with other medical disciplines as possible means to obtain better results.

A partnership with anesthetists interested in the field of interventional cancer pain management has been highlighted as a key to achieve successful cancer pain control [6, 7]. Combination of conventional medical management with intraspinal drug delivery resulted in significant pain relief, improved side effect profile, and a trend towards prolonged survival [8]. Despite being previously suggested in the literature, the role of invasive procedures in the management of cancer pain traditionally remains a last resort [9] and is not being regarded as part of a collaboration between different medical disciplines.

To our knowledge the efficacy of interdisciplinary approaches combining nursing and different medical specialties such as palliative care, anesthesia, and radiooncology has never been reported.

*The MUHC Cancer Pain Clinic*. The Cancer Care Mission of the MUHC serves over 1500 new cancer patients per year within the province of Quebec. Prior to the creation of the MUHC Cancer Pain Clinic (CPC), ambulatory patients with poorly controlled cancer pain were referred either to a chronic pain unit (the Alan Edwards Pain Management Unit) or to a palliative care clinic (MUHC Palliative Care Day Hospital). With time, it became obvious that a closer collaboration between these two departments could lead to better management of challenging cancer pain cases. The CPC was created in 2011 with the goal of offering an interdisciplinary approach to assess and manage cancer pain. The clinical team meets three mornings a week to do new patient assessments and follow-ups and the cancer pain nurse follows up with the patients and coordinates the care via scheduled and ad hoc phone calls from Monday to Friday. The great majority of patients are referred from the department of oncology. These referrals are triaged to the CPC when pain is the most prevalent symptom. At their first visit, patients are assessed simultaneously by a palliative care physician, a nurse clinician specialist in oncology and palliative care, and an anesthesiologist specialized in interventional pain procedures. The team is completed by a radiation oncologist joining the CPC once a week. Before meeting the patient the team reviews the case to obtain details about cancer status, current symptoms, and ongoing treatment. Patients are then seen at the same time with all the specialists present in the room. Upon finishing the assessment, a brief case discussion takes place for obtaining a clinical consensus and deciding the analgesic plan. When deemed necessary, other treatments such as physiotherapy, occupational therapy, and psychosocial support are offered upon referral. Ambulatory treatment continues with frequent scheduled and ad hoc phone consultations with the cancer pain nurse to monitor initial response to treatments, evaluate new symptoms, or answer queries patients may have. At all times, treating oncologists and the cancer pain team remain in contact to update the cancer status, coordinating the care, avoiding contraindications with ongoing antineoplastic therapies, and anticipating and preventing changes in the pain severity secondary to cancer treatments. Patients are seen in clinic usually 4 weeks after initial assessment (FU_1_). The next assessment (FU_2_) is done normally within the subsequent 6 ± 2 weeks depending on the patients' response to treatments.

The effectiveness of the care provided by the CPC interdisciplinary team was assessed with a program evaluation completed at the end of the first year of operation. Of 200 new patients seen in the first two years of activity, a pain relief of 2.3 points measured with the average pain item of the Brief Pain Inventory questionnaire was observed between assessment and the second follow-up (data not published). These initial positive results prompted the design of a retrospective analysis focused on the description of the treatment modalities provided and detailed analysis of patient-reported outcomes such as pain, disability, and other cancer-related symptoms.

## 2. Methods

### 2.1. Design

A retrospective chart review of ambulatory patients seen at the CPC between April 2013 and March 2014 was conducted. The study was approved by the institutional ethics board and conducted in accordance with good clinical practice and applicable Canadian regulatory requirements.

### 2.2. Participants

Charts were included in the review provided that (a) patients had been assessed and then seen as follow-up at the CPC at least twice, (b) patients had completed all the clinical questionnaires provided in clinic, and (c) the medical chart included the appropriate data to complete the research questionnaire.

### 2.3. Data Collection

Research data were collected from the medical charts at three study time points: baseline (initial assessment) and the two subsequent follow-ups (FU_1_ and FU_2_). Variables recorded included demographic data (age and gender), cancer status (primary tumour and staging), and the analgesic therapy. The analgesic plan could include pharmacological treatments (acetaminophen, nonsteroid anti-inflammatory drugs, steroids, opioids, antidepressants, anticonvulsants, atypical antipsychotics, sedatives, and/or synthetic cannabinoids) and nonpharmacological approaches including interventional procedures, radiation therapy, physiotherapy, occupational therapy, and/or psychosocial counselling.

#### 2.3.1. Patients Follow-Up

Patients were asked to complete the Edmonton Symptom Assessment System (ESAS) [10] at every visit and the Brief Pain Inventory (BPI) questionnaire [11] upon initial assessment and at FU_2_. Pain severity was classified as mild (0–3), moderate (4–7), or severe (8–10) [12].

Patients were considered responders when their pain severity, measured by ESAS-pain and BPI-worst pain diminished beyond 30% or 50% at FU_2_.

Additionally, calculation of morphine equivalent daily doses (MEDD) was done separately for short and long acting opioids [13] with the exception of methadone. At the CPC, we favour opioid consumption in long acting formulations to increase efficacy, improve compliancy, and decrease side effects, hence our interest in separating the MEDD calculation between long and short acting agents.

### 2.4. Statistical Analysis

Descriptive statistics including the mean and standard deviation for continuous variables and the count and proportion for categorical variables were produced. Changes over time from baseline in pain severity and other symptoms were assessed for statistical significance using the paired-samples *t*-test. Independent predictors of response were assessed using multivariate logistic regression. Parameters considered as potential predictors were those showing a statistical trend (*p* < 0.150) in univariate analysis and the parsimonious model was derived using backwards variable selection at a *p* < 0.05 level.

## 3. Results

From a total of 186 patients seen as new patients, 71 fulfilled the inclusion criteria and were included in the analysis. Main reasons for exclusion were lack of completion of clinical questionnaires (38.6%), poorly documented analgesic plan in the chart (27.7%), patients seen only once or twice in clinic before being referred to another service (25.5%), and patients referred to the CPC only for consideration of interventional cancer pain approaches (8.2%).

### 3.1. Patient Characteristics

Data on demographics and oncological status is summarized in Table 1. Of interest, two-thirds of patients presented with advanced cancer disease (locally advanced or metastatic) upon initial assessment.

### 3.2. Pain Severity

**Figure 1:**
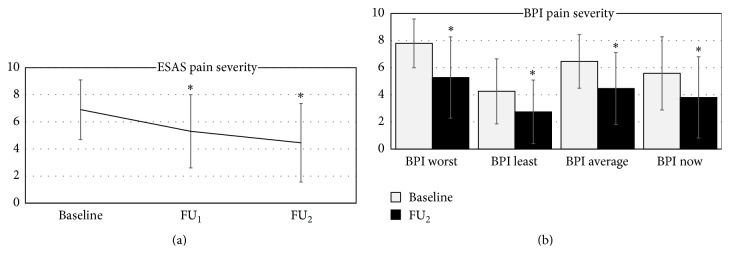
((a) and (b)) Pain severity along the course of the study. Statistically significant and clinically meaningful reductions were observed between baseline and FU_2_ in all pain questionnaires rating pain intensity (ESAS *p* = 0.0000001; BPI worst *p* = 0.00000006; BPI least *p* = 0.0005; average pain *p* = 0.00004; pain now *p* = 0.0006). Values depict average and standard deviation. ^*∗*^Paired  *t*-tests compared with baseline, *p* value < 0.05.

Pain was assessed with the first question of the ESAS questionnaire and the first four items of the BPI questionnaire. Figures 1(a) and 1(b) depict the pain severity at baseline (BL) and during follow-ups. A significant reduction in pain severity was consistently reported in all pain items during the two follow-up visits compared to baseline.

The absolute pain relief calculated as the raw difference of BPI average pain between BL and FU_2_ was 2 points on the 0–10 NRS.

**Figure 2:**
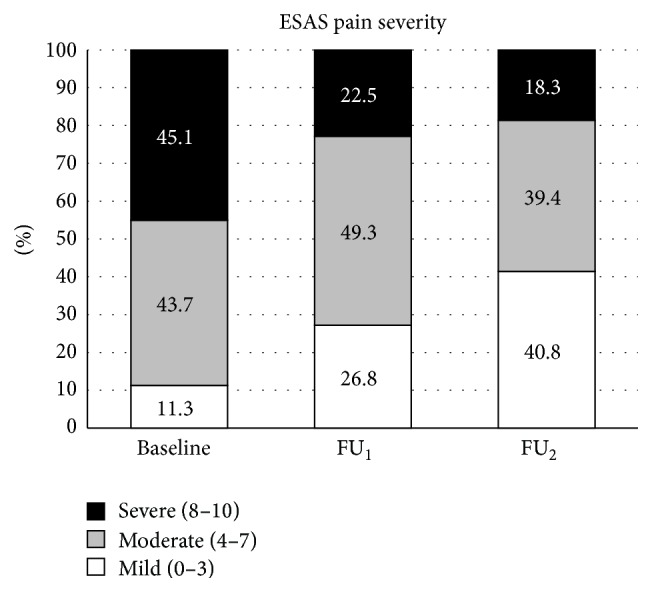
Distribution of patients by pain categories.

Analysis of pain severity by categories (Figure 2) demonstrated a shift over time in the pattern of patients with mild, moderate, and severe pain. Whereas 45% of patients were experiencing severe pain upon initial assessment, this percentage dropped to 18% at FU_2_. The percentage of patients with mild pain increased from 11% to 40% after initial treatment, showing improvement from BL.

A positive response to the therapy was considered in patients presenting with a pain reduction of 30% or 50% at FU_2_, using the ESAS and the BPI worst pain. The percentage of responders was more than half of the sample when the threshold was set at 30% relief. Setting a more stringent threshold of >50% pain reduction produced a lower, yet substantial percentage of more than one-third of the sample (Table 2).

Thirteen patients (18% of the total population) presented with unchanged or increased pain severity at FU_2_. This subpopulation of nonresponders presented with a similar prevalence of advanced disease (61%) and the most common cancer site was gastrointestinal and gynecological. Interestingly, all of them received opioid therapy during the treatment but none methadone. More than half of them (61%) received at least one nonpharmacological therapy.

### 3.3. Relief of Other Symptoms

The severity of symptoms other than pain also decreased after the course of initial treatment. Reductions in the severity of all symptoms, except appetite and shortness of breath, reached statistical significance (see Table 3).

### 3.4. Disability

Pain-related disability investigated with the BPI questionnaire also decreased. Comparison between BL and FU_2_ demonstrated a significant reduction in all disability ratings except for mood and normal work. A composite interference scale of the BPI as an index of clinically significant improvement was also found significantly different between BL and FU_2_ (Table 4).

### 3.5. Therapies Offered during Initial Course of Cancer Pain Treatment

An overview of the treatment modalities offered to patients is outlined in Tables 5 and 6.

*(a) Pharmacological Treatment*. Upon initial consultation, the most common drugs prescribed were (in descending order) short acting opioids (76.1%), long acting opioids (43.7%), acetaminophen (42.3%), and anticonvulsants (25.4%). At FU_2_ the proportion changed: most common were long acting opioids (59.1%) followed by short acting opioids and acetaminophen (56.3% each). Overall, the percentage of patients taking analgesics of every category increased except for short acting opioids which decreased by 20%.

**Figure 3:**
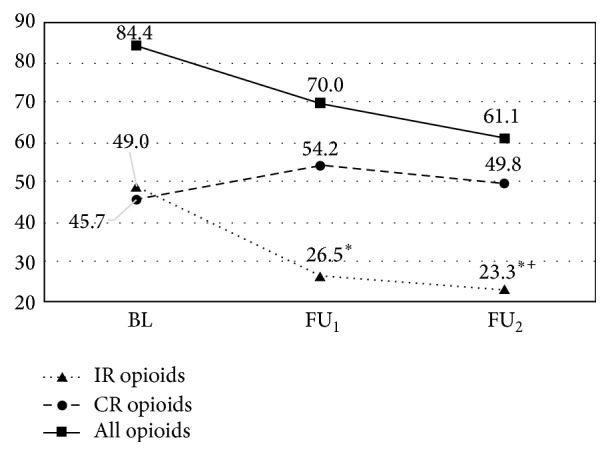
Average morphine equivalent daily dose (mg/day). ^*∗*^Comparison with baseline. Paired-samples *t*-test *p* < 0.005. ^+^Comparison between IR (immediate release) and CR (controlled release) opioids. Independent-samples *t*-test *p* < 0.005.

A calculation of morphine equivalent daily dose (MEDD) was performed separately for short acting and long acting opioid (Figure 3) excluding methadone, whose equivalence ratio with morphine is variable [14]. A significant reduction in the doses of short acting opioids consumed at FU_1_ and FU_2_ was observed. The mean dose of long acting opioids also changed during the study yet these changes were not statistically significant.

The comparison between short and long acting opioids revealed a change in consumption patterns throughout the study. At baseline, patients were essentially taking the same amount of short and long acting formulations whereas at FU_2_ most prescribed opioids were controlled release preparations.

Ten patients (14.1%) were taking methadone for pain relief during their first assessment. These patients were normally referred to the CPC after initial consultation with supportive and palliative care department during a recent hospitalization. Upon discharge, these patients were scheduled to be followed at CPC for further cancer pain treatment. At FU_1_ 13 patients (18.3%) were receiving methadone with a further increase to 17 patients at FU_2_ (23.9%).

*(b) Nonpharmacological Analgesic Therapies*. 57% of patients received at least one of the five nonpharmacological analgesic options (Table 6). The most common approach was interventional therapy in 28% of patients. The most common (38%) anesthetic procedures were peripheral or radicular nerve procedures (i.e., intercostal cryoneurolysis, lumbar radicular pulsed radiofrequency neuromodulation). The second most common (33%) anesthetic procedures involved sympathetic anatomical structures (i.e., splanchnic, celiac, superior hypogastric plexus or ganglion impar neurolysis with phenol).

The second most common nonpharmacological approach was psychosocial therapy in 18% of cases.

### 3.6. Predictors of Positive Analgesic Outcomes

An exploratory multivariate analysis was conducted in order to identify independent predictors of positive analgesic outcomes over the follow-up period defined as pain relief beyond 30% or 50% using the ESAS pain and the worst pain BPI (Table 7). Male gender was consistently found to be a significant predictor for positive analgesic response to the treatment associated with a 3.5- to 6-4-fold increase in the odds of achieving each target as compared to females.

In addition, opioid use at baseline was associated with significantly higher odds (OR [95% CI]: 4.8 [1.1, 21.7]) of achieving 30% improvement in ESAS pain, while use of radiotherapy was a significant predictor of poor outcome for 50% improvement in ESAS pain (OR [95% CI]: 0.1 [0.02, 0.9]). No significant predictors of 50% improvement in BPI worst pain were identified. Correlation analysis to identify predictors of response to the treatment is presented in Table 8. A univariate analysis was done, crossing pain relief at FU_2_ beyond 30% or 50% using the ESAS pain and the worst pain BPI. Across the different pain questions, only male gender was found consistently to be a significant predictor for positive analgesic response to treatment.

## 4. Discussion

The cancer pain approach described in this study adopted the multidisciplinary team work as a model, taking it one step further by having multiple pain specialists simultaneously interacting with the patient. This is the foundation of an interdisciplinary approach, defined as “a synthesis of two or more disciplines, establishing a new level of disclosure and integration of knowledge” [15]. The logic evolution of this effort is towards transdisciplinarity, where holistic schemes look at the dynamic of the whole system by subordinating disciplines. The results of the current retrospective study indicate that this approach resulted in decreased pain and improved function.

### 4.1. Pain Scores and Other Cancer-Related Symptoms

A significant reduction in pain severity was consistently reported across the two follow-up visits. This difference not only reached statistical significance but can be considered clinically meaningful [16].

A responder was the patient presenting with at least 30% reduction of pain. This threshold is considered clinically meaningful in other pain trials [17], yet a 30% reduction can possibly be explained by a robust placebo response. A stricter threshold of 50% was thus selected still yielding satisfactory results in one-third of our patient population.

Other cancer-related symptoms decreased as well after the initial course of treatment but did not reach statistical significance in the case of appetite and shortness of breath. This may be explained by the fact that we did not use the last version of the ESAS, the ESAS revised [18]. This version corrects potentially misleading questions involving appetite. The lack of significant relief of shortness of breath could be explained by a lack of correlation between shortness of breath and other cancer-related symptoms reported in previous studies [19].

Pain-related disability decreased at FU_2_. This reduction was statistically significant for most but not all items. Changes in mood, normal work, and enjoyment of life did not reach statistical significance. If a two-point difference or a 30% reduction is selected as a minimum for clinically important difference [20], well-being was the only item that reached an important improvement. A change in a composite score of all ratings on the ESAS and BPI can be interpreted as change in overall rating of disease burden or quality of life [21]. The significant reduction observed at FU_2_ can be interpreted as improvement in quality of life despite the presence of advanced cancer disease.

### 4.2. Therapeutic Options

Study patients received opioid and nonopioid analgesic medications. The use of coadjuvants has been clearly recommended in the management of cancer pain [22]. In the cohort of the present report the use of anticonvulsants and antidepressants was higher than reported in cancer pain patients [23]. The combination of coadjuvants and nonpharmacological interventions has been shown to be beneficial not only for improved analgesia but also for its potential opioid sparing effect [24]. We could not demonstrate an obvious opioid sparing effect in our patient population; however, a significant reduction of the total amount of short acting opioids taken between baseline and the two subsequent follow-up visits was found. To the authors, it is noteworthy that patients were taking less than half the amount of short acting opioids at FU_2_ compared with their initial baseline intake.

### 4.3. Predictors

Several factors have been identified in the literature as predictors of pain relief in cancer patients. These include improvement in concomitant depression, higher socioeconomic status, and fewer comorbid conditions. Patients with severe pain at baseline and with recurrent or progressive cancer disease were less likely to experience pain improvement [25]. In an exploratory analysis, we tried conducting a similar analysis in our population but male gender was the only variable consistently identified as a significant independent predictor of positive analgesic outcomes. We could not find any correlation between tumour site and pain responders; therefore, this may reflect inherent differences in pain assessment between genders as previously shown in several studies [26, 27]. Opioid use at baseline and radiotherapy were also identified as positive and negative predictors of analgesic outcome, respectively.

It is possible that additional factors may be significantly associated with cancer pain relief; however they may have not been able to be identified due to the low sample size of our study. Additional studies with larger sample sizes are required to extensively characterize the predictors of cancer pain relief.

### 4.4. Limitations

Less than half of new patients seen during one year did not fulfill the inclusion criteria. Most of these patients did not complete the clinical questionnaires or the analgesic plan was not fully clear from the medical chart. The second main reason for not including these patients in the analysis was that they were not seen at least three consecutive times at the CPC. This is a frequent occurrence in our CPC since our patient population presents with advanced cancer disease associating important morbidity. It is our clinical routine to transfer the case to the supportive and palliative care day hospital when the disease burden becomes too important. Other limitations of the current study are inherent to the retrospective chart review design, including concerns with regard to the internal validity of the findings due to the potential incompleteness of the information collected and the lack of a comparator group. Creating such a control group within our McGill University Health Centre has become challenging since the CPC has become the main ambulatory unit treating cancer pain patients, making it unlikely to find another group of advanced cancer pain patients being treated with a different approach.

## 5. Conclusion

The MUHC interdisciplinary approach to assess and manage cancer pain provides effective relief of pain and other cancer-related symptoms, associated with a reduction of functional impairment and an improvement of symptom burden. This approach combines pharmacological and nonpharmacological analgesic therapies along with patient-centered care to provide personalised treatment for each case. Further prospective trials are warranted to provide stronger evidence of this approach.

## Figures and Tables

**Table 1:** Demographic and cancer status.

Age: mean ± SD	62.9 ± 12	
Gender *n* (%)	♀ 37 (52%); ♂ 34 (48%)	
Cancer status *n* (%)	Local 24 (33.8%) versus advanced 47 (66.2%)	

Cancer site	*N* (%)	% of advanced cases

Gastrointestinal	17 (23%)	65%
Bronchus and lung	14 (19.7)	79%
Breast	7 (9.9%)	57%
Head and neck	7 (9.9)	29%
Haematological	7 (9.9%)	29%
Gynecological	6 (8.5%)	29%
Urological	6 (8.5%)	100%
Musculoskeletal	3 (4.2%)	100 %
Others	4 (5.6%)	100%

**Table 2:** Percentage of responders comparing baseline and FU_2_.

Category	% of responders
ESAS > 30% relief	53.5%
ESAS > 50% relief	36.6%
BPI (worst pain) > 30% relief	44.2%
BPI (worst pain) > 50% relief	36.1%

**Table 3:** Other symptoms ratings as per ESAS.

Symptom	Baseline	FU_1_	FU_2_
Pain	6.9 ± 2.2	5.3 ± 2.2^*∗*^	4.5 ± 2.9^*∗*^
Fatigue	6.3 ± 2.7	5.6 ± 2.7^*∗*^	5.2 ± 3.0^*∗*^
Nausea	2.7 ± 3.1	1.7 ± 2.5^*∗*^	1.9 ± 2.7^*∗*^
Depression	3.1 ± 3.3	2.0 ± 2.7^*∗*^	2.2 ± 2.8^*∗*^
Anxiety	4.1 ± 3.3	2.6 ± 2.6^*∗*^	2.7 ± 3.0^*∗*^
Drowsiness	4.7 ± 2.9	3.2 ± 2.7^*∗*^	3.3 ± 3.2^*∗*^
Appetite	5.0 ± 2.2	6.9 ± 2.2	6.9 ± 2.2
Well-being	6.9 ± 3.0	4.3 ± 2.8^*∗*^	4.3 ± 2.8^*∗*^
Shortness of breath	3.3 ± 3.2	2.8 ± 3.1	3.3 ± 3.1
ESAS total score	40.3 ± 16.4	31.3 ± 15.1^*∗*^	30.9 ± 17.4^*∗*^

^*∗*^Paired *t*-tests compared with baseline, *p* value < 0.05.

**Table 4:** Pain interference.

Pain interference	Baseline	FU_2_
General activity	7.1 ± 2.6	5.3 ± 2.9^*∗*^
Mood	5.8 ± 2.9	4.8 ± 3.1
Walking ability	5.9 ± 2.9	4.8 ± 3.3^*∗*^
Normal work	6.9 ± 3.1	6.1 ± 3.0
Relations with others	5.4 ± 2.9	4.0 ± 3.3^*∗*^
Sleep	6.0 ± 3.2	4.3 ± 3.4^*∗*^
Enjoyment of life	6.2 ± 3.5	4.7 ± 3.4
Well-being	6.9 ± 3.0	4.3 ± 2.8^*∗*^
BPI interference composite score	39.1 ± 14.9	27.0 ± 16.8^*∗*^
BPI total score	61.7 ± 19.9	41.2 ± 24.3^*∗*^

^*∗*^Paired *t*-tests compared with baseline, *p* value < 0.05.

**Table 5:** Analgesics drugs offered at the Cancer Pain Clinic.

	Recorded at baseline (%)	Recorded at FU_2_ (%)
Tylenol	42.3	56.3
NSAID	16.9	28.2
Steroids	9.9	19.7
Anticonvulsants	25.4	39.4
Antidepressants	14.1	25.4
Antipsychotics	5.6	16.9
Sedatives	14.1	22.5
Cannabinoids	2.8	5.6
Short acting opioids	76.1	56.3
Long acting opioids	43.7	59.1
Methadone	14.1	21.1

**Table 6:** Nondrug analgesic interventions offered at the Cancer Pain Clinic.

Interventional therapy	28.2%
Psychosocial interventions	18.3%
Radiotherapy	12.7%
Physiotherapy	11.3%
Occupational therapy	4.2%

**Table 7:** Predictors for positive analgesic outcomes.

Variables predicting a pain reduction (*p* < 0.05)	
50% BPI worst pain	Use of methadone at FU_2_ Use of short acting opioids at FU_2_
30% BPI worst pain	Male gender
50% ESAS pain	Male gender
30% ESAS pain	Male gender Using any opioid during the initial visit Prescription of sedatives at FU_1_

**Table 8:** Predictors of positive analgesic outcomes.

Variable	30% ESAS pain			50% ESAS pain			30% BPI worst pain			50% BPI worst pain^†^		
	OR	95% CI	*p* value	OR	95% CI	*p* value	OR	95% CI	*p* value	OR	95% CI	*p* value
Sex (male versus female)	6.4	1.7, 23.6	*0.005*	3.7	1.3, 10.6	*0.015*	3.5	1.1, 10.9	*0.030*	—	—	—
NSAIDs initiated in clinic (yes versus no)	—	—	—	5.2	0.8, 33.9	0.086	4.7	0.8, 29.5	0.095	—	—	—
Methadone use at baseline (yes versus no)	—	—	—	—	—	—	0.1	0.01, 1.4	0.093	—	—	—
Opioid use at baseline (yes versus no)	4.8	1.1, 21.7	*0.039*	—	—	—	—	—	—	—	—	—
Radiotherapy use in clinic (yes versus no)	—	—	—	0.1	0.02, 0.9	*0.035*	—	—	—	—	—	—

Final multivariate model after variable selection using *p*_In_ = 0.05 and *p*_Out_ = 0.100. Variables considered in the model were those showing a *p* value of <0.150 in univariate logistic regression. Potential predictors tested were gender (male versus female), age, site of primary tumour, disease type (localized versus advanced), BPI worst pain at baseline, separate use of analgesics (acetaminophen, NSAID, steroids, antiepileptic, antidepressants, antipsychotics, sedatives, cannabinoids, opioids, or methadone), and indication of nondrug analgesic interventions (radiotherapy, psychotherapy, physiotherapy, occupational therapy, and interventional therapy) during the course of treatment.

^†^No independent predictors were identified for 50% improvement in BPI worst pain.

Statistically significant variables are highlighted in italics.
